# Socioeconomic determinants of childhood obesity among primary school children in Guangzhou, China

**DOI:** 10.1186/s12889-016-3171-1

**Published:** 2016-06-08

**Authors:** Weijia Liu, Wei Liu, Rong Lin, Bai Li, Miranda Pallan, K. K. Cheng, Peymane Adab

**Affiliations:** Faculty of School Health, Guangzhou Centre for Disease Control and Prevention, Guangzhou, China; Public Health, Epidemiology and Biostatistics, Institute of Applied Health Research, College of Medical and Dental Sciences, University of Birmingham, Birmingham, UK

**Keywords:** Socioeconomic status, Obesity, School children, China

## Abstract

**Background:**

Socioeconomic inequalities in childhood obesity prevalence differ according to a country’s stage of nutrition transition. The aim of this study was to determine which socioeconomic factors influence inequalities in obesity prevalence in Chinese primary school children living in an urban setting.

**Methods:**

We assessed obesity prevalence among 9917 children aged 5–12 years from a stratified random sample of 29 state-funded (residents) and private (migrants) schools in Guangzhou, China. Height and weight were objectively measured using standardised methods and overweight (+1 SD < BMI-for-age z-score ≤ +2 SD) and obesity (BMI-for-age z-score > +2 SD) were defined using the World Health Organisation reference 2007. Socioeconomic characteristics were ascertained through parental questionnaires. Generalised Linear Mixed Models with schools as a random effect were used to compare likelihood of overweight/obesity among children in private, with public schools, adjusting for child age and sex, maternal and paternal BMI and education level, and household per-capita income.

**Results:**

The prevalence of overweight/obesity was 20.0 % (95 % CI 19.1 %–20.9 %) in resident compared with 14.3 % (95 % CI 13.0 %–15.4 %) in migrant children. In the adjusted model, the odds of overweight/obesity remained higher among resident children (OR 1.36; 1.16–1.59), was higher in boys compared with girls (OR 2.56; 2.24–2.93), and increased with increasing age (OR 2.78; 1.95–3.97 in 11–12 vs 5–6 year olds), per-capita household income (OR 1.27; 1.01–1.59 in highest vs lowest quartile) and maternal education (OR 1.51; 1.16–1.97 in highest vs lowest). Socioeconomic differences were most marked in older boys, and were only statistically significant in resident children.

**Conclusions:**

The socioeconomic gradient for childhood obesity in China is the reverse of the patterns seen in countries at more advanced stages of the obesity epidemic. This presents an opportunity to intervene and prevent the onset of social inequalities that are likely to ensue with further economic development. The marked gender inequality in obesity needs further exploration.

## Background

Globally, the increasing prevalence of obesity is a major public health problem in both the developed and the developing world, and is widely recognised as a key risk factor for a range of chronic diseases [1]. In China, the prevalence of overweight and obesity among school-aged children has increased more rapidly and over a shorter time period than that seen in most Western countries [2, 3], with prevalence in some urban regions approaching those in developed countries [4, 5]. Studies across China have also highlighted accelerating childhood obesity rates in rural areas, which face a double burden of over and undernutrition [6].

Developed countries which are at a more advanced stage of the obesity epidemic are facing a widening socioeconomic gap in childhood obesity [7]. Obesity prevalence is increasing among children in lower socioeconomic groups, whilst prevalence is stabilising or decreasing among children from more advantaged backgrounds [8]. However developing countries tend to have the reverse pattern, with higher childhood obesity prevalence among higher socioeconomic groups [9]. A few studies from China suggest that this trend may be changing in urban areas [10].

Rapid economic growth in China has resulted in major rural–urban migration [11]. Over 20 million people per year move out of rural areas [12], so that migrants now comprise a high proportion (up to 50 %) of the population in major urban cities in China [13]. In general, migrants tend to have lower levels of education and lower income [14]. To our knowledge, previous studies have not examined obesity rates among children from migrant families, compared to city residents in urban centres in China, nor examined whether the socioeconomic differences in childhood obesity are common to both groups. In China, there are two main types of primary schools. The majority of schools are state-funded public primary schools, which are managed by local government and funded mostly from taxation, with strict residency-based enrollment policies. The other are privately run primary schools owned by individuals or social organizations and funded mostly from tuition fees. Children of migrant families tend to attend the privately run, rather than state-funded public schools. This provides an opportunity to access children from a variety of backgrounds for assessing obesity patterns.

It is important to establish where China currently sits in terms of the obesity epidemic, in order to target preventive interventions where they are most needed, and also to prevent the onset of health inequalities that are widening in the developed world. Furthermore, it is important to disentangle which aspects of socioeconomic status (e.g. migrant status, income or parental education) are the main drivers of inequalities in childhood obesity. Previous studies have focused on differences in childhood obesity in urban versus rural areas, or across income groups within urban centres. Here, we estimated the prevalence of childhood obesity in a major city in China, comparing prevalence by migrant status and taking into account parental education and income.

## Methods

### Participants and setting

We conducted a cross-sectional analysis of data collected between April and June in 2014, of 9917 children aged 5–12 years from primary schools in Guangzhou, China. Guangzhou is a leading commercial southern Chinese city with a population of about 12.9 million, around a third of whom are migrant households [15]. There is public provision of schooling for primary school aged children whose parents are city residents, whereas children of migrant families attend private schools.

A multi-stage stratified cluster random sampling method was used to obtain a representative sample. Using a random number generator, five of the ten urban districts were first selected. Within each of the selected districts, schools were stratified by public (residents) or private (migrant) status, and six primary schools were randomly chosen, with a 2:1 ratio from each stratum. Within each school two classes per year group (from grade 1 to 5) were randomly selected, from which all pupils were invited to take part. Permission for the study was not obtained for one of the private schools in our sampling frame, leaving 29 participating schools.

### Measurements and questionnaires

Informed consent for measurements was sought from the parents of all eligible children, and verbal assent was obtained from the children at the time of measurement. Anthropometric and blood pressure measurements and physical fitness assessments were undertaken by trained research staff, using standardised procedures and instruments. Parents were also asked to complete a questionnaire, detailing sociodemographic information and child lifestyle habits. In this paper, sociodemographic and anthropometric measurement data were used and are described in more detail below.

### Anthropometric measurements

Height and weight were measured with subjects wearing light clothing and without shoes. Height was recorded to the nearest 0.1 cm with a TGZ type height tester (Dalian). Weight was measured to the nearest 0.1 kg using an electronic scale (JH-1993 T, weighing Apparatus Co.Ltd. Dalian). Body mass index (BMI; [weight (kg)]/[height (m)]^2^), was calculated and BMI standard deviation scores (BMI z-score) were derived using the age (calculated by subtracting the date of birth from the date of examination) and sex specific WHO growth reference for school-aged children [16], which were further classified as non-overweight (≤1SD), overweight (>1SD) and obese (>2SD).

### Parent questionnaires

The parent questionnaire included information on parents’ household income, number of people living in the household, and maternal and paternal educational level. Annual household income was divided by household size to obtain per-capita income, which was further categorised into quartiles (<12,000RMB, 12,001–20,000RMB, 20,001-34286RMB, ≥34287RMB). Parental education was categorised into low (primary and junior high school level), medium (senior high and vocational school level) or high (university level or higher). In addition, self-reported height and weight for each mother and father was obtained, from which parental BMI was derived. This was used to classify parents into the following weight status categories: normal weight (BMI < 24), overweight (BMI ≥ 24 < 28), obese (BMI ≥ 28), as defined by the guideline for prevention and control of overweight/obesity in Chinese adults [17].

### Statistical analysis

Data were analysed using SPSS 21.0 statistical software package (SPSS Inc., Chicago, IL). Descriptive analysis was used to describe participant characteristics and the prevalence of overweight and obesity. Bivariate analysis was used to compare the prevalence of overweight/obesity by sociodemographic factors. In addition Pearson’s correlation was used to examine the relationships between child and both maternal, and paternal BMI. Generalised linear mixed models were used to compare likelihood of overweight/obesity among children in private (migrant status), with public (resident status) schools, adjusting for child age and sex, maternal and paternal BMI and education level, and household per-capita income, with school as a random effect. We also described the prevalence of overweight/obesity by category of per-capita annual income for each age group, in boys compared with girls, and in migrant compared with non-migrant groups. Finally, we carried out sensitivity analyses: a) using the Chinese national reference norm introduced by the working group on obesity in China (WGOC), and b) the criteria introduced by international obesity taskforce (IOTF), instead of the WHO 2007 child growth standard. A 2-tailed P value less than 0.05 was considered statistically significant.

## Results

### Characteristics of the study sample

Among eligible children, 86.6 % (9917/11445) agreed to take part in the study, resulting in a final sample of 9917 children aged 5–12 years, from 29 schools (9 private; 3057 children). The characteristics of the study population are summarised in Table 1. Boys were over-represented in the study sample, particularly in the migrant population (male:female ratio 1:42) and there was a higher proportion of older children (11–12 years) among migrant, compared with resident populations. Resident children were more likely to live in households with higher income and to have parents with higher levels of education, compared with migrants. Overall, 18.2 % (95%CI 17.4 %–19.0 %) of all children were overweight or obese, with the proportion being higher among resident (20.0 %; 95%CI 19.1–20.9 %) compared with migrant (14.3 %; 95 % CI 13.0–15.4 %) children. Overweight/obesity was higher among fathers of resident compared to migrant children (41.5 % and 36.9 % respectively), whereas the reverse was true for mothers (17.7 % and 20.1 % respectively).

**Table 1:** Participant characteristics stratified by child’s household registration

Characteristics	Total	Migrant students	Non-Migrant (Resident) students	
	Mean ± SD or N (%)	Mean ± SD or N (%)	Mean ± SD or N (%)	*P* value
N	9917	3057 (30.8)	6860 (69.2)	
Age (years)	9.24 ± 1.50	9.27 ± 1.60	9.24 ± 1.45	0.243
BMI z-score	–0.19 ± 1.31	–0.29 ± 1.22	–0.14 ± 1.34	< 0.001
Gender				
Boys	5529 (55.8)	1792 (58.6)	3737 (54.5)	< 0.001
Girls	4388 (44.2)	1265 (41.4)	3123 (45.5)	
Age				
5–6 years	657 (6.6)	242 (7.9)	415 (6.0)	< 0.001
7–8 years	3784 (38.2)	1116 (36.5)	2668 (38.9)	
9–10 years	3942 (39.7)	1178 (38.5)	2764 (40.3)	
11–12years	1534 (15.5)	521 (17.0)	1013 (14.8)	
Level of yearly income (per-capital)				
1st quartile (≤12000 RMB)	1463 (17.3)	669 (27.4)	772 (13.0)	< 0.001
2nd quartile (12001–20000 RMB)	2327 (27.6)	882 (34.9)	1445 (24.4)	
3rd quartile (20001–34286 RMB)	2545 (30.2)	609 (24.1)	1936 (32.7)	
4th quartile (≥ 34287 RMB)	2105 (24.9)	342 (13.5)	1763 (29.8)	
Father’s education				
Primary school and junior high school	3919(42.6)	1858 (67.9)	2061 (31.9)	< 0.001
Senior high school and vocational school	4006(43.5)	783 (28.6)	3223 (49.8)	
University and above	1281(13.9)	96 (3.5)	1185 (18.3)	
Mother’s education				
Primary school and junior high school	4608(50.0)	2103 (76.9)	2505 (38.6)	< 0.001
Senior high school and vocational school	3551 (38.5)	565 (20.7)	2986 (46.1)	
University and above	1057 (11.5)	66 (2.4)	991 (15.3)	
Child Weight status				
Normal weight	8112 (81.8)	2621 (85.7)	5491 (80.0)	< 0.001
Overweight	1126 (11.4)	290 (9.5)	836 (12.2)	
Obese	679 (6.8)	146 (4.8)	534 (7.8)	
Father’s Weight status				
Normal weight	5037 (59.8)	1534 (63.0)	3503 (58.5)	0.001
Overweight	2853 (33.9)	764 (31.4)	2089 (34.9)	
Obese	529 (6.3)	135 (5.5)	394 (6.6)	
Mother’s Weight status				
Normal weight	7034 (81.6)	1975 (79.9)	5059 (82.3)	0.003
Overweight	1394 (16.2)	425 (17.2)	969 (15.8)	
Obese	190 (2.2)	72 (2.9)	118 (1.9)	

Note: There are some missing vales in the analysis variables above. Analysis Variables: Level of yearly income (1477); Father’s education (711); Mother’s education (701); Father’s weight status (1498); Mother’s occupation (1299). 1^st^quartile: 1 %–25 % percentiles, 2^nd^quartile: 26 %–50 % percentiles, 3^rd^quartile: 51 %–75 % percentiles, 4^th^quartile: 76 %–100 % percentiles

### Socio-demographic factors associated with childhood overweight/obesity

The relationship between socio-demographic factors and children’s weight status is summarized in Table 2. Overweight/obesity prevalence increased with increasing age (9.1 % in 5–6 years compared with 22.0 % in 11–12 years) and was higher in boys (23.5 %) compared with girls (11.6 %). There was also a statistically significant moderate relationship between child and both maternal and paternal BMI (r = 0.21 and 0.18 respectively, *p <* 0.05).

**Table 2:** Generalized linear mixed model analysis of socioeconomic factors correlates of overweight and obesity among school children in Guangzhou, China

Characteristics	Overweight or obese		Likelihood of overweight/obesity	
	Number (%)		Crude OR (95 % CI)	Adjusted OR (95 % CI)
Gender				
Girls	4388 (11.6) ^**^	Reference		Reference
Boys	5529 (23.5)	2.40 (2.15–2.69)^**^		2.56 (2.24–2.93) ^**^
Age				
5–6 years	657 (9.1) ^**^	Reference		Reference
7–8 years	3784 (16.2)	1.88 (1.42–2.49) ^**^		1.95 (1.39–2.74) ^**^
9–10 years	3942 (20.1)	2.46 (1.86–3.25) ^**^		2.59 (1.85–3.62) ^**^
11–12 years	1534 (22.0)	2.77 (2.06–3.71) ^**^		2.78 (1.95–3.97) ^**^
Household per-capita annual income				
1st quartile (≤12000 RMB)	1463 (14.8) ^*^	Reference		Reference
2nd quartile (12001–20000 RMB)	2327 (16.3)	1.07 (0.89–1.28)		1.01 (0.82–1.24)
3rd quartile (20001–34286 RMB)	2545 (19.1)	1.22 (1.02–1.46) ^*^		1.21 (0.99–1.49) ^*^
4th quartile (≥34287 RMB)	2105 (21.4)	1.34 (1.11–1.63) ^*^		1.27 (1.01–1.59) ^*^
Father’s education				
Primary school and junior high school	3919 (16.2)	Reference		Reference
Senior high school and vocational	4006 (20.0)	1.13 (0.99–1.27)		1.11 (0.95–1.31)
University and above	1281 (19.8)	1.01 (0.84–1.21)		0.89 (0.69–1.16)
Mother’s education				
Primary school and junior high school	4608 (16.0) ^*^	Reference		Reference
Senior high school and vocational	3551 (20.1)	1.19 (1.05–1.35) ^*^		1.21 (1.03–1.43) ^*^
University and above	1057 (22.8)	1.32 (1.10–1.59) ^*^		1.51 (1.16–1.97) ^*^
Father’s weight status				
Non-overweight	5037 (14.9) ^**^	Reference		Reference
Overweight	2853 (22.9)	1.67 (1.48–1.87) ^**^		1.63 (1.43–1.85) ^**^
Obese	529 (31.2)	2.54 (2.08–3.11) ^**^		2.48 (1.98–3.11) ^**^
Mother’s weight status				
Non-overweight	7034 (15.9) ^**^	Reference		Reference
Overweight	1394 (29.3)	2.27 (1.98–2.59) ^**^		2.41 (2.07–2.80) ^**^
Obese	190 (30.5)	2.56 (1.86–3.52) ^**^		2.51 (1.72–3.67) ^**^
Type of school				
Private (migrants)	3057(14.3) ^**^	Reference		Reference
Public (residents)	6860 (20.0)	1.48 (1.19–1.83) ^**^		1.36 (1.16–1.59) ^**^

Note: Generalised Linear Mixed Models were used for multivariateanalyses, adjusted model includes all social-demographic factors above, and school as a random effect. Dependent variable: children were categorized into two groups (overweight/obese and non-overweight) using WHO 2007. OR, odds ratio; CI, confidence interval. 1^st^quartile: 1 %–25 % percentiles, 2^nd^quartile: 26 %–50 % percentiles, 3^rd^quartile: 51 %–75 % percentiles, 4^th^quartile: 76 %–100 % percentiles.*p < 0.05, **p < 0.001

The likelihood of overweight/obesity increased with increasing quartile of household per-capita income and with increasing maternal education. There was no significant relationship between paternal education levels and child weight status. In the multilevel model adjusted for child age and sex, parental weight status, parental education level, and household per capita annual income, resident, compared with migrant children, had significantly higher odds of being overweight/obese.

### Relationship between income category and weight status by age group and migrant status

**Fig. 1:**
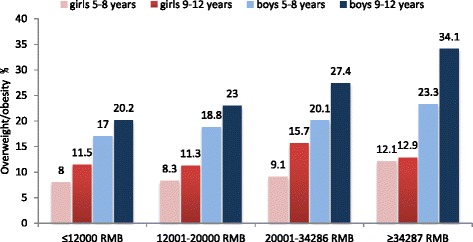
Prevalence of overweight/obesity among younger (age 5–8 years) and older (age 9–12 years) Chinese primary school boys and girls according to quartiles of per capita household income

Figure 1 shows the prevalence of overweight/obesity among younger and older boys and girls, according to annual per-capita household income. For all income groups, obesity prevalence was statistically significantly higher among boys compared with girls and in older compared with younger children. However, the sex disparity in obesity prevalence widened with increasing household income, and the age disparity was most marked among boys from the highest income households.

**Fig. 2:**
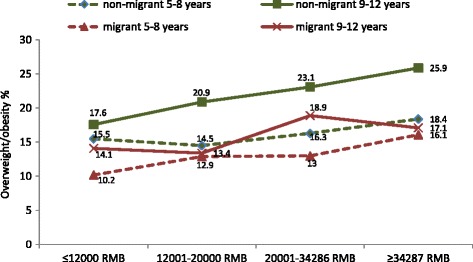
Prevalence of overweight/obesity among younger (age 5–8 years) and older (age 9–12 years) resident and migrant Chinese primary school children according to quartiles of per capita household income

Figure 2 compares the relationship between household per-capita income and prevalence of overweight/obesity by migrant status in two age groups. Among children from resident households, there is a clear relationship between increasing household income and increased prevalence of obesity, particularly in the older age group (*p <* 0.05). However, there is no statistically significant relationship between household income and obesity prevalence among children from migrant households.

### Sensitivity analyses

Repeating the analyses for factors associated with overweight/obesity, using the WGOC and IOTF instead of the WHO 2007 reference standard, did not alter the findings. The magnitude and direction of effect of all variables reported above remained similar to the main analysis, although the absolute values differed very slightly.

## Discussion

We found that the prevalence of obesity among primary school children in an urban setting in China varied greatly according to sociodemographic characteristics. Overall, rates were highest among boys compared with girls, and increased with age, household per-capita income and higher maternal education levels. Social inequalities in obesity were particularly seen in boys, and widened with age. To our knowledge, this is the first study that has examined childhood obesity prevalence in urban migrants and contrasted this with prevalence in urban residents. In the adjusted models, household income, maternal education, and migrant status were all independently associated with obesity. However, among children from migrant households, overweight rates did not differ much between income groups, suggesting that social inequalities have not yet emerged within migrant families.

The overall prevalence of obesity in primary school children is similar to those reported in other studies in urban centres in China [18, 19] and the gender [20, 21] and socioeconomic [20] disparity are also in keeping with previous studies. Overweight/obesity rates among preadolescent boys (aged 9–12 years) in the highest income households were over 34.1 %, which is similar to the prevalence in many developed countries [22–25]. In contrast, the prevalence of obesity in pre-adolescent girls (~12 %), was lower than rates in the West and did not differ as much between high and low income groups. The positive socioeconomic gradient in obesity among boys and girls is opposite to that observed in developed countries, and may be due to the current stage of economic development in China [20]. Affordability of high energy dense foods and access to more sedentary modes of transport and leisure pursuits is likely to be greater among higher income families. We found that independently of household income, higher maternal education was also associated with higher rates of obesity. This is contrary to findings in the West, where higher parental education is associated with lower childhood obesity [26, 27] and suggests that factors other than purchasing power also contribute to the socioeconomic gradient. Maternal education has been shown to be associated with better child nutrition and growth internationally, with lower obesity in developed countries, and with lower rates of stunting and malnutrition in rural China [28].

The marked gender difference in obesity also contrasts with the pattern observed in most other countries [29]. The reason for lower rates of obesity among girls is not clear, but may be due to societal norms of ideal body image for girls [30, 31] or a cultural preference for boys [32], leading to overfeeding. The latter may also explain the higher male to female ratio in school pupils observed in this study.

Including a large representative sample of children in a typical urban setting in China with objective measures of adiposity and adjustment for parental weight status are strengths of this study. Furthermore, use of more than one measure of socioeconomic status and comparison of migrant with resident households within the same setting offer novel insights. The main limitation is the cross-sectional nature, which does not allow us to infer causal relationships. Furthermore the measures of family income, parents’ education and parents’ weight status were all based on self-report, which may reduce their validity. We also did not have data on duration of residency in Guangzhou among migrant families, and so were not able to examine differences according to time since migration.

## Conclusions

Our findings suggest that contrary to findings in some previous studies [6], a reversal in the socioeconomic gradient for childhood obesity has not yet occurred in our study population. There is an opportunity to apply lessons from countries at more advanced stage of the obesity epidemic to prevent the shift in obesity patterns and subsequent social inequalities that are likely to ensue with further economic development. Rural–urban migrants, and those with lower household income and education have lower rates of obesity, which could be maintained with the introduction of government food and physical activity policies [33–35]. The marked gender inequality in obesity needs further exploration.

## Abbreviations

CI, confidence intervals; OR, odds ratio; SD, standard deviation; BMI, body mass index; WGOC, working group on obesity in China; IOTF, international obesity taskforce; WHO, world health organization
